# Analysis of State Medicaid Expansion and Access to Timely Prenatal Care Among Women Who Were Immigrant vs US Born

**DOI:** 10.1001/jamanetworkopen.2022.39264

**Published:** 2022-10-28

**Authors:** Teresa Janevic, Ellerie Weber, Frances M. Howell, Morgan Steelman, Mahima Krishnamoorthi, Ashley Fox

**Affiliations:** 1 Blavatnik Family Women’s Health Research Institute, New York, New York; 2 Department of Obstetrics, Gynecology and Reproductive Science, Icahn School of Medicine at Mount Sinai, New York, New York; 3 Department of Population Health Science and Policy, Icahn School of Medicine at Mount Sinai, New York, New York; 4 Department of Medical Education, Icahn School of Medicine at Mount Sinai, New York, New York; 5 Johns Hopkins School of Medicine, Baltimore, Maryland; 6 Rockefeller College of Public Affairs and Policy, University at Albany, SUNY, Albany, New York

## Abstract

**Importance:**

Disparities exist in access to timely prenatal care between immigrant women and US-born women. Exclusions from Medicaid eligibility based on immigration status may exacerbate disparities.

**Objective:**

To examine changes in timely prenatal care by nativity after Medicaid expansion.

**Design, Setting, and Participants:**

A cross-sectional difference-in-differences (DID) and triple-difference analysis of 22 042 624 singleton births from January 1, 2011, to December 31, 2019, in 31 states was conducted using US natality data. Data analysis was performed from February 1, 2021, to August 24, 2022.

**Exposures:**

Within 16 states that expanded Medicaid in 2014, the rate of timely prenatal care by nativity in years after expansion was compared with the rate in the years before expansion. Similar comparisons were conducted in 15 states that did not expand Medicaid and tested across expansion vs nonexpansion states.

**Main Outcomes and Measures:**

Timely prenatal care was categorized as prenatal care initiated in the first trimester. Individual-level covariates included age, parity, race and ethnicity, and educational level. State-level time-varying covariates included unemployment, poverty, and Immigrant Climate Index.

**Results:**

A total of 5 390 814 women preexpansion and 6 544 992 women postexpansion were included. At baseline in expansion states, among immigrant women, 413 479 (27.3%) were Asian, 110 829 (7.3%) were Black, 752 176 (49.6%) were Hispanic, and 238 746 (15.8%) were White. Among US-born women, 96 807 (2.5%) were Asian, 470 128 (12.1%) were Black, 699 776 (18.1%) were Hispanic, and 2 608 873 (67.3%) were White. Prenatal care was timely in 75.9% of immigrant women vs 79.9% of those who were US born in expansion states at baseline. After Medicaid expansion, the immigrant vs US-born disparity in timely prenatal care was similar to the preexpansion level (DID, –0.91; 95% CI, –1.91 to 0.09). Stratifying by race and ethnicity showed an increase in the Asian vs White disparity after expansion, with 1.53 per 100 fewer immigrant women than those who were US born accessing timely prenatal care (95% CI, –2.31 to –0.75), and in the Hispanic vs White disparity (DID, –1.18 per 100; 95% CI, –2.07 to –0.30). These differences were more pronounced among women with a high school education or less (DID for Asian women, –2.98; 95% CI, –4.45 to –1.51; DID for Hispanic women, –1.47; 95% CI, –2.48 to –0.46). Compared with nonexpansion states, differences in DID estimates were found among Hispanic women with a high school education or less (triple-difference, –1.86 per 100 additional women in expansion states who would not receive timely prenatal care; 95% CI, –3.31 to –0.42).

**Conclusions and Relevance:**

The findings of this study suggest that exclusions from Medicaid eligibility based on immigration status may be associated with increased health care disparities among some immigrant groups. This finding has relevance to current policy debates regarding Medicaid coverage during and outside of pregnancy.

## Key Points

**Question:**

Were there changes in immigrant vs US-born disparities in timely prenatal care after Medicaid expansion owing to immigrant eligibility exclusions?

**Findings:**

In this cross-sectional difference-in differences analysis including 2.4 million women preexpansion vs 6.5 million postexpansion, overall population findings were null. Among Asian and Hispanic subgroups in only expansion states, access to timely prenatal care increased from 2011 to 2019 in US-born but not immigrant women, resulting in an increased immigrant vs US-born disparity.

**Meaning:**

The findings of this study suggest that exclusions from Medicaid eligibility based on immigration status may increase maternal health care disparities in some immigrant populations.

## Teaser

This cross-sectional study examines timely access to prenatal care among women who are immigrants vs those who are US born before and after Medicaid expansion.

## Introduction

In the US, 1 in every 4 births is to a mother who is an immigrant.^1^ Yet, immigrants who are noncitizens are excluded from critical safety-net programs that low-income US citizens are able to access, including health care through Medicaid.^2^ Expansion of Medicaid has filled gaps in maternal health coverage and some evidence points to improved maternal health in states that have expanded Medicaid.^3^ However, Medicaid eligibility exclusions for some categories of immigrants may result in their exclusion from the positive effects of the Medicaid expansion.

Timely prenatal care is critical because it allows for early screening, intervention, and health education that may benefit mothers and infants beyond birth.^4^ Increasing the proportion of women receiving early and adequate prenatal care is a goal of Healthy People 2030.^5^ Increased insurance coverage before pregnancy is thought to improve timely prenatal care because women who have a regular source of medical care are better positioned to receive pregnancy testing and an appointment with a pregnancy health care professional more quickly.^6^ Insurance coverage outside of pregnancy may also increase access to family planning, and women with planned pregnancies are more likely to access timely prenatal care.^7^ More generous Medicaid eligibility is associated with increased insurance coverage before pregnancy, and insurance coverage during that period is associated with earlier entry to prenatal care.^8^ Therefore, nonpregnancy Medicaid coverage is an important lever to improve timely prenatal care.

A concern is that some state Medicaid policies, which can exclude persons based on immigration status, discriminately harm the health of mothers who are immigrants and their infants. In many states, even expansion states, immigrants who are unauthorized, are legal permanent residents, or have legal status for less than 5 years are not eligible for Medicaid. Approximately 23% of immigrants in the US are thought to be unauthorized, 5% are temporary lawful residents, 27% are legal permanent residents but not citizens, and 45% are naturalized citizens, so the potential influence of these exclusions on population health is large.^9^ Only 5 states allow coverage for nonpregnant adult legal permanent residents, and only the District of Columbia allows coverage for unauthorized nonpregnant individuals who are immigrants (eTable 1 in the Supplement).^10^ It is unknown how these policy exclusions affect the health of mothers who are immigrants, despite the fact that they make up a sizeable proportion of US deliveries. Identifying health outcomes of Medicaid policy toward immigrant women may help direct state and national health policies to address these gaps, while also providing valuable evidence for health care and social services professionals trying to fill these gaps through other resources.

Using US natality data for the years 2011-2019, our objective was to estimate the association between exclusion from nonpregnancy Medicaid coverage and access to prenatal care in immigrant women. We hypothesized that in states that expanded Medicaid in 2014 as part of the Affordable Care Act, the rate of timely prenatal care would increase in US-born women but not those who were immigrants, creating a left-behind effect. We also conducted stratified analyses by race and ethnicity to learn the potential outcome of Medicaid exclusions on racial and ethnic maternal health equity. We hypothesized the left-behind effect would be greatest in women without a college education.

## Methods

### Data Source

We obtained January 1, 2011, to December 31, 2019, US natality data from the National Center for Health Statistics. The Program for the Protection of Human Subjects at Icahn School of Medicine at Mount Sinai determined this research exempt from review and the requirement for indformed consent because the data did not include identifiers. This study followed the Strengthening the Reporting of Observational Studies in Epidemiology (STROBE) reporting guideline for reporting cross-sectional studies.

In 2003, the US birth certificate underwent a revision that changed the collection of several key variables used in our research, which was henceforth adopted by states in different years. Therefore, only 37 reporting areas (36 states plus the District of Columbia) that transitioned to the revised birth certificate by 2010 were eligible for inclusion. Of 29 108 274 births for 2011 to 2019 in these 37 areas, we limited the sample to singleton births (n = 28 119 442) (eFigure 1 in the Supplement). We then excluded women younger than 20 years (n = 1 794 403) because they may have been eligible for the Children’s Health Insurance Program. Because a major focus of this study was racial and ethnic subgroup analysis, we next excluded those who reported a racial or ethnicity category classified as other (n = 782 884) (described in the Exposure Identification and Additional State Exclusions section). We also excluded those for whom the birthplace was unknown (n = 63 112). Next, we excluded records with any missing data (n = 1 015 995 [4%]); we chose this approach because missing birth certificate data likely cannot satisfy the assumption of missing-at-random needed for imputation. We did not include late expanders to reduce model complexity and so that all states would have equal follow-up periods. We also excluded the District of Columbia because it provided Medicaid to individuals who were undocumented immigrants before 2014. The resulting sample of births in 31 states was 22 042 624.

### Gender Inclusion

National Center for Health Statistics birth data do not include information on gender identity, and we use the terms *mother*, *woman*, and *maternal* to be consistent with the terminology in the data source. However, to be inclusive of all pregnancy-capable genders, we consider the terms to apply to any person who is pregnant or capable of being pregnant or who has delivered a child.

### Outcome

Our outcome of interest was timely prenatal care. We used the variable month prenatal began and categorized women who began prenatal care during the first trimester as timely prenatal care = 1 or timely prenatal care = 0. We combined late and no prenatal care because having no prenatal care was rare in our sample (1.5%). In 2014 the National Center for Health Statistics transitioned its method of calculating gestational age of newborns to the obstetric estimate of gestation at delivery from previous use of the last normal menses.^11^ This change also affected the calculation of trimester of prenatal care and resulted in a small increase across all states. Our analytic approach was robust to this small increase because it is equal across groups.

### Exposure Identification and Additional State Exclusions

We classified individuals born outside of the US to be immigrant. We used mother’s Hispanic origin and mother’s race recode to create the categories non-Hispanic Asian, non-Hispanic Black, Hispanic, and non-Hispanic White (henceforth, Asian, Black, Hispanic, and White). Our sample comprised 31 states, 16 of which expanded Medicaid eligibility in 2014 and 15 that did not (eTable 1 in the Supplement).^12^ The resulting expansion states sample for the primary analysis was 11 935 806 births.

### Covariates

Individual-level covariates included maternal age, maternal educational level, and parity. We included state-level time-varying covariates plausibly associated with timely prenatal care for which co-occurring trends may have occurred: state unemployment rate, state poverty rate, and the Immigrant Climate Index.^13,14^ The Immigrant Climate Index comprises city, county, and state laws that provide either a benefit or restriction on immigrant life and has been associated with very preterm birth in Hispanic women.^15^ Using data from the Urban Institute, we examined whether there were any changes in Medicaid policies toward immigrants concurrent with the Medicaid expansion and found no changes (eTable 1 in the Supplement). All regressions included state- and year-level fixed effects.

### Statistical Analysis

Data analysis was performed from February 1, 2021, to August 24, 2022. We graphically examined trends in timely prenatal care by nativity, race and ethnicity, and state expansion status. We also examined graphs for evidence that the preintervention parallel trends assumption holds, as required for our difference-in-differences (DID) approach. We tested an interaction term for year by nativity in the preexpansion period in all racial and ethnic categories combined and in racial and ethnic subgroups to further evaluate the parallel trends assumption.

We estimated DID coefficients using a linear probability model and the resulting marginal predicted probabilities.^16^ We calculated a robust SE that accounted for within-state clustering.^17^ The DID regressions included main effect coefficients for postexpansion vs preexpansion and immigrant women vs US born, and the interaction of the 2 variables. We used a 1-year lag period, because the mechanism by which nonpregnancy Medicaid may affect timely prenatal care would occur before conception. We stratified by race and ethnicity to obtain effect estimates for Asian, Black, Hispanic, and White women. We reran models using a subset of mothers with an educational level of a high-school degree or less. We expected that women in this group would be more likely to be left behind, because those with a lower educational level are more likely to meet income eligibility criteria for Medicaid. For all subgroups we estimated 2 models: adjusted for maternal health characteristics and additionally adjusted for state unemployment and poverty rates, as well as the Immigrant Climate Index. Next, we reran models in nonexpansion states. We then used triple difference (DDD) models to formally test the difference in findings between expansion and nonexpansion states. The rationale for DDD models is that evidence that differences in immigrant vs US-born disparities are larger in expansion compared with nonexpansion states would strengthen the inference that any left-behind effect in expansion states is due to expansion and not to other unmeasured causes.

We ran the regressions using a logit probability model instead of a linear probability model. Following the literature, we tried omitting Wisconsin, as well as including it as an expansion state; although Wisconsin has not expanded Medicaid as part of the Affordable Care Act, it expanded offering Medicaid to all childless adults up to 100% federal poverty level as of April 1, 2014.^18,19^ We also tried specifications that included New Hampshire and the District of Columbia, specifications that included fewer and no time-varying state characteristics, and specifications that included a linear time trend. We conducted a leave-one-out analysis. All analyses were conducted in Stata, version 14.2 (StataCorp LLC). With 2-sided, unpaired testing, findings were considered significant at α < .05.

## Results

In expansion states, there were a total of 5 390 814 women preexpansion and 6 544 992 women postexpansion. At baseline in expansion states, 413 479 (27.3%) immigrant women were Asian, 110 829 (7.3%) were Black, 752 176 (49.6%) were Hispanic, and 238 746 (15.8%) were White (Table 1). In contrast, 96 807 (2.5%) of US-born women were Asian, 470 128 (12.1%) were Black, 699 776 (18.1%) were Hispanic, and 2 608 873 (67.3%) were White. Immigrant women were in the youngest age group (14.9%), compared with 25.9% of those born in the US. In addition, 28.7% of immigrant women had less than a high school education compared with 8.7% of women born in the US. The distribution of sociodemographic characteristics in expansion states changed negligibly postexpansion; the same is true across race and ethnicity (eTables 3-6 in the Supplement) and for nonexpansion states (eTable 2 in the Supplement).

**Table 1.** Sample Characteristics in Expansion States

Characteristic	No. (%)			
	Preexpansion (2011-2014)		Postexpansion (2015-2019)	
	Immigrant (n = 1 515 230)	US born (n = 3 875 584)	Immigrant (n = 1 787 516)	US born (n = 4 757 476)
Race and ethnicity				
Asian	413 479 (27.3)	96 807 (2.5)	520 560 (29.1)	122 147 (2.6)
Black	110 829 (7.3)	470 128 (12.1)	150 570 (8.4)	562 810 (11.8)
Hispanic	752 176 (49. 6)	699 776 (18.1)	808 843 (45.2)	965 214 (20.3)
White	238 746 (15.8)	2 608 873 (67.3)	307 543 (17.2)	3 107 305 (65.3)
Age, y				
20-24	225 347 (14.9)	1 004 662 (25.9)	217 801 (12.2)	1 023 703 (21.5)
25-29	437 150 (28.9)	1 191 722 (30.7)	471 380 (26.4)	1 148 150 (30.9)
30-34	480 068 (31.7)	1 093 007 (28.2)	604 017 (33.8)	1 430 951 (30.1)
35-39	292 055 (19.3)	477 867 (12.3)	383 502 (21.5)	696 608 (14.6)
40-54	80 610 (5.3)	108 326 (2.8)	110 816 (6.2)	138 064 (2.9)
Multiparous				
Yes	1 012 377 (66.8)	2 366 163 (61.1)	1 177 641 (65.9)	2 950 868 (62.0)
Education				
<High school	435 552 (28.7)	337 489 (8.7)	406 461 (22.7)	353 011 (7.4)
High school	332 954 (22.0)	902 958 (23.3)	397 014 (22.2)	1 137 370 (23.9)
Some college	286 789 (18.9)	1 330 424 (34.3)	346 095 (19.4)	1 560 006 (32.8)
Bachelor’s/graduate degree	459 935 (30.4)	1 304 713 (33.7)	637 946 (35.7)	1 707 089 (35.9)

Women born in the US had higher rates of timely prenatal care than those who were immigrants across the study period (Figure). Timely prenatal care increased in all racial and ethnicity categories combined, and in Asian and Black immigrant women. Timely prenatal care decreased after expansion in Hispanic immigrant women. In nonexpansion states, trends in timely prenatal care differed by racial and ethnic subgroup (eFigure 2 in the Supplement). Visually inspecting preintervention trends suggested reasonably parallel trends in expansion states in immigrant women and those who were US born, whereas, in nonexpansion states, trends diverged in some groups (Figure; eFigure 2 in the Supplement). An interaction term for year by nativity in the preintervention period, however, was significant, suggesting nonparallel trends, for Black and Asian women in both expansion and nonexpansion states and for White women in nonexpansion states.

**Figure.**
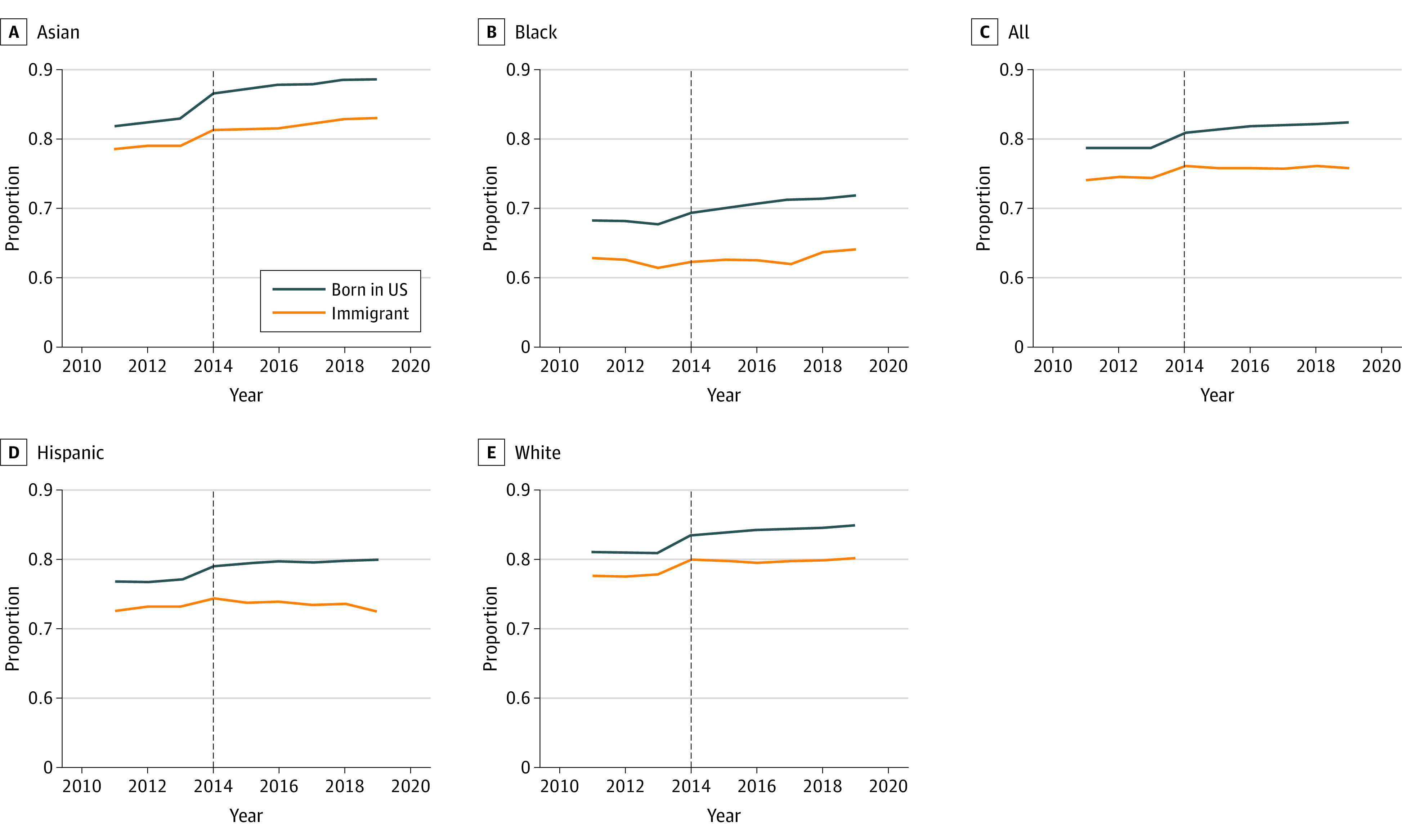
Trends in Timely Prenatal Care by Race and Ethnicity and Nativity Among Expansion States, 2011-2019

In all racial and ethnicity categories combined in expansion states, 75.9% of immigrant women and 79.9% of those who were US born received timely prenatal care at baseline (Table 2). In fully adjusted analyses, preexpansion, 3.48 fewer per 100 immigrant women received timely prenatal care compared with US-born women (95% CI, −6.95 to −0.01); postexpansion in these same states, the disparity grew to 4.39 fewer (95% CI, −7.07 to −1.71; DID, −0.91, 95% CI, −1.91 to 0.09). Among Asian women, 1.53 per 100 fewer immigrant women vs US born received timely prenatal care (95% CI, −2.31 to −0.75). Among Hispanic women, 1.18 per 100 fewer immigrant women received timely prenatal care (95% CI, −2.07 to −0.30). No pre- vs postdifferences in disparities were found among the Black and White races (DID for Black women, −1.47; 95% CI, −3.58 to 0.65; DID for White women, −0.28; 95% CI, −1.49 to 0.94). Results were more pronounced among women with lower educational levels (Table 3). DID estimates showed 2.98 per 100 fewer Asian immigrant women (95% CI, −4.45 to −1.51) and 1.47 per 100 fewer Hispanic immigrant women (95% CI, −2.48 to −0.46) had timely prenatal care after expansion compared with their US-born counterparts. Results for nonexpansion states were mostly null (eTable 7 and eTable 8 in the Supplement).

**Table 2.** Rate of Timely Prenatal Care Pre– and Post–Medicaid Expansion in Expansion States

Race and ethnicity	Preexpansion			Postexpansion			Difference-in-difference, No. per 100 (95% CI)
	Immigrant, %	US born, %	Adjusted difference, No. per 100 (95% CI)^a^	Immigrant, %	US born, %	Adjusted difference, No. per 100 (95% CI)^a^	
All	75.9	79.9	−3.48 (−6.95 to −0.01)	77.9	82.7	−4.39 (−7.07 to −1.71)	−0.91 (−1.91 to 0.09)
Asian	80.4	84.6	−3.57 (−4.65 to −2.5)	83.3	89.8	−5.11 (−5.63 to −4.58)	−1.53 (−2.31 to −0.75)
Black	61.8	69.7	−10.71 (−13.89 to −7.53)	62.8	72.2	−12.18 (−14.82 to −9.53)	−1.47 (−3.58 to 0.65)
Hispanic	74.7	78.1	−0.77 (−3.37 to 1.84)	76.3	81.1	−1.95 (−3.87 to −0.03)	−1.18 (−2.07 to −0.30)
White	78.5	82.0	−5.63 (−7.88 to −3.38)	80.8	84.8	−5.91 (−7.99 to −3.83)	−0.28 (−1.49 to 0.94)

^a^ Adjusted for race and ethnicity, age, parity, educational level, state unemployment rate, state poverty rate, and Immigrant Climate Index, with difference adjusted for immigrant vs US-born status.

**Table 3.** Rate of Timely Prenatal Care Pre– and Post–Medicaid Expansion in Expansion States Among Women With High School Education or Less

Race and ethnicity	Preexpansion			Postexpansion			Difference-in-difference, No. per 100 (95% CI)
	Immigrant, %	US born, %	Adjusted difference, No. per 100 (95% CI)^a^	Immigrant, %	US born, %	Adjusted difference, No. per 100 (95% CI)^a^	
All	71.8	70.3	−1.76 (−5.69 to 2.18)	73.3	73.6	−2.78 (−5.79 to 0.23)	−1.03 (−2.35 to 0.29)
Asian	70.7	69.4	0.31 (−2.77 to 3.39)	75.7	78.4	−2.67 (−4.84 to −0.50)	−2.98 (−4.45 to −1.51)
Black	57.0	63.3	−7.64 (−10.90 to −4.37)	57.9	66.1	−9.35 (−11.57 to −7.13)	−1.71 (−4.40 to 0.97)
Hispanic	73.3	73.0	0.2 (−2.82 to 3.22)	74.6	76.4	−1.27 (−3.64 to 1.10)	−1.47 (−2.48 to −0.46)
White	69.4	71.2	−3.84 (−6.75 to −0.93)	72.2	74.4	−4.24 (−6.59 to −1.89)	−0.4 (−2.36 to 1.56)

^a^ Adjusted for race and ethnicity, age, parity, educational level, state unemployment rate, state poverty rate, and Immigrant Climate Index, with difference adjusted for immigrant vs US-born status.

When testing whether the DID coefficients differed between expansion and nonexpansion states using DDD regression, we found differences only among Hispanic women (Table 4). An additional 1.41 per 100 Hispanic immigrant women were left behind US-born Hispanic women postexpansion in expansion states compared with nonexpansion states (95% CI, −2.89 to −0.07). The difference was larger within women with lower educational levels (DDD, −1.86; 95% CI, −3.31 to −0.42).

**Table 4.** Comparison of Difference-in-Differences for Timely Prenatal Care Between Medicaid Expansion and Nonexpansion States Using DDD

Race and ethnicity	Overall sample		High school sample	
	DDD (95% CI)^a^	No.	DDD (95% CI)^a^	No.
Full sample	−0.09 (−1.81 to 1.62)	22 042 624	−1.19 (−3.20 to 0.81)	8 217 696
Asian	0.16 (−0.85 to 1.17)	1 561 927	1.02 (−4.14 to 2.10)	312 817
Black	3.46 (0.28 to 6.63)	2 967 657	2.76 (−0.31 to 5.83)	1 341 786
Hispanic	−1.41 (−2.89 to −0.07)	5 772 138	−1.86 (−3.31 to −0.42)	3 454 064
White	1.97 (−1.13 to 5.07)	11 740 902	1.20 (−2.75 to 5.15)	3 109 029

Abbreviation: DDD, triple differences.

^a^ Adjusted for race and ethnicity, age, parity, educational level, state unemployment rate, state poverty rate, and Immigrant Climate Index.

Alternative model specifications produced similar coefficients and robustness checks and sensitivity analyses did not appreciably change results. A leave-one-out-analysis for Hispanic women in expansion states revealed no influential states, with the exception of California, which when omitted moderately reduced the effect estimate from −1.18 per 100 to −0.50 per 100 Hispanic women.

## Discussion

We found persistent disparities in access to timely prenatal care in immigrant women compared with those who were US born from 2011 to 2019. In states that expanded Medicaid as part of the Affordable Care Act, increasing access to timely prenatal care among US-born women, but not among Asian and Hispanic immigrant women, resulted in an increase in the disparity in timely prenatal care. The results were most pronounced among Hispanic and Asian women without a college degree. This left-behind effect remained after controlling for time-varying state-level poverty, unemployment, and policies toward immigrants.

Our findings should be considered in the context of the existing literature on state and federal policy and maternal health care use among women who are immigrants. Previous research has reported that state expansion of public health insurance specific to women who are pregnant and immigrants resulted in improved access to prenatal care,^20,21,22^ as well as improved detection of comorbidities and pregnancy complications.^22^ Our findings suggest that the opposite outcome may occur when expansions of nonpregnancy Medicaid programs exclude persons based on immigration status. Current literature examining the influence of nonpregnancy Medicaid expansion on maternal health care use in the overall population has mixed findings.^23,24,25,26,27,28^ Our analysis builds on this research with a new focus on a large proportion of birthing individuals not eligible for Medicaid in most areas in the US.

Our finding that Medicaid exclusions appear to be detrimental to prenatal care use by women who are immigrants is a key link to emerging evidence on maternal health disadvantage faced by women who are immigrants.^29^ The healthy immigrant effect is often found in research on reproductive health, such that immigrant women fared better than their US-born counterparts.^30^ A 2018 report noted that, in fact, this health advantage erodes over time^31^ and is not apparent in all immigrant groups.^32^ Moreover, some women who are immigrants have poorer maternal outcomes compared with those who are US born, for example, higher rates of preeclampsia,^33^ gestational diabetes,^34,35^ and severe maternal morbidity.^36^ Exclusionary policies as a form of structural racism—the interaction between institutional, systemic, and social forces that reinforces inequities among certain racial and ethnic groups^37^—are under scrutiny as factors affecting these emerging perinatal health disadvantages.^38^

A notable finding in nonexpansion states was that timely prenatal care decreased throughout the study period among Black immigrant women. This lack of parallel preintervention trend meant we could not conduct a reliable DDD analysis, but the decrease trend observed is concerning. It is possible that a synergistic effect of racism and anti-immigrant rhetoric resulted in decreased timely prenatal care among Black immigrant women. The reluctance to use public services or benefits to which one has a right due to fear of negative consequences is known as the chilling effect.^39^ The chilling effect may occur due to fear of reprisal owing to documentation status or the public charge rule that stipulates that acceptance of certain safety-net benefits may affect the legal path to citizenship for a person who is an immigrant. Anti-immigrant rhetoric was found to be associated with an increase in delayed prenatal care in Houston.^40^ Routine monitoring of Healthy People 2030 indicators by race and ethnicity and nativity could alert medical professionals to similar concerning factors.

### Limitations

This study has limitations. First, a concern exists about the lack of preintervention parallel trends in some subgroups. Although no one test for the parallel trend assumption is considered a standard, visual inspection appeared to indicate that this condition may not be satisfied in all subgroups, particularly in the nonexpansion states. However, the parallel trend assumption appeared to be met among Hispanic women in both expansion and nonexpansion states, and this phenomenon may explain why our findings regarding Hispanic women most robustly supported our hypothesis. Next, our results could be influenced by a migration cohort effect. For example, the Black immigrant population has both increased and shifted geographically from 2010 to 2019.^41^ Any unmeasured sociodemographic difference in groups arriving to the US during the period of study could potentially explain differences. Another limitation is that other state-level policies or maternal health programming existing in Medicaid expansion states only may have improved access to timely prenatal care differentially among US-born individuals for reasons other than citizenship-related exclusions. Such policies, for example, could explain why our leave-one-out analysis found slightly attenuated results when California was omitted. In addition, we did not have information on immigration status of individuals; instead, we used country of birth as a proxy for immigrant status. We therefore were unable to exclude from our analysis individuals who would be eligible for Medicaid, including US citizens born outside of the US. The proportion of undocumented individuals does not vary greatly between states and is estimated to be between 20% and 40% of the immigrant population in most states.^9^ Therefore, our reported effect estimates of 1.0 to 1.5 per 100 persons being left behind at the population level in expansion states are likely underestimates of the magnitude of the result among excluded populations (eg, undocumented women).

This study has implications for a range of policies denying benefits to persons based on immigration status. Currently, legislation to extend pregnancy Medicaid coverage in the postpartum period is being considered in many states.^42^ Policy makers and advocates should be aware that exclusion of persons based on citizenship could have a profound influence. For example, undocumented immigrants or those with legal permanent residence of less than 5 years are not eligible for some relief measures meant to buffer the economic impact of the COVID-19 pandemic.^43^ Arguments exist against citizen-based exclusions to health care and social benefits based on a human rights framework^44^; our work suggests the population-level influence on the fifth of birthing individuals in the US who are foreign-born.

## Conclusions

This study has implications for a range of policies denying benefits to persons based on immigration status. Currently, legislation to extend pregnancy Medicaid coverage in the postpartum period is being considered in many states.^42^ Policy makers and advocates should be aware that exclusion of persons based on citizenship could have a profound influence. For example, undocumented immigrants or those with legal permanent residence of less than 5 years are not eligible for some relief measures meant to buffer the economic impact of the COVID-19 pandemic.^43^ Arguments exist against citizen-based exclusions to health care and social benefits based on a human rights framework^44^; our work suggests the population-level influence on the fifth of birthing individuals in the US who are foreign-born.

In this study, we noted sizeable disparities between women who were immigrant vs US born in access to timely prenatal care, and these disparities were exacerbated in some immigrant groups after Medicaid expansion. Structural racism faced by immigrants, such as exclusionary health policies, must be addressed to achieve maternal health equity.

## Notes

Supplement. **eFigure 1.** Flowchart of Study Sample Inclusion and Exclusion Criteria **eTable 1.** Medicaid Nonpregnancy and Pregnancy Coverage for Immigrants Pre- and Post-Medicaid Expansion in Expansion and Nonexpansion States in Study Sample **eFigure 2.** Trends in Timely Prenatal Care by Race and Ethnicity and Nativity in Medicaid Nonexpansion States, 2011-2019 **eTable 2.** Sample Characteristics by Nativity, Nonexpansion States, Pre- and Post- Medicaid Expansion **eTable 3.** Sample Characteristics by Nativity Among Asian Women in Expansion Women in Expansion States Pre- and Post-Medicaid Expansion **eTable 4.** Sample Characteristics by Nativity Among Black Women in Expansion Women in Expansion States Pre- and Post-Medicaid Expansion **eTable 5.** Sample Characteristics by Nativity Among Hispanic Women in Expansion Women in Expansion States Pre- and Post-Medicaid Expansion **eTable 6.** Sample Characteristics by Nativity Among White Women in Expansion Women in Expansion States Pre- and Post-Medicaid Expansion **eTable 7.** Rate of Timely Prenatal Care Pre- and Post-Medicaid Expansion in Nonexpansion States, by Nativity and Race and Ethnicity, 2011-2019 **eTable 8.** Rate of Timely Prenatal Care Pre- and Post-Medicaid Expansion in Nonexpansion States, by Nativity and Race and Ethnicity, Among Women With High School Education or Less, 2011-2019
