# Supplementary material for: Analysis of State Medicaid Expansion and Access to Timely Prenatal Care Among Women Who Were Immigrant vs US Born
Source: JAMA Netw Open. 2022 Oct 28;5(10):e2239264. doi: 10.1001/jamanetworkopen.2022.39264 (PMC9617172; doi:10.1001/jamanetworkopen.2022.39264)
Supplement: Supplement. — eFigure 1. Flowchart of Study Sample Inclusion and Exclusion Criteria eTable 1. Medicaid Nonpregnancy and Pregnancy Coverage for Immigrants Pre- and Post-Medicaid Expansion in Expansion and Nonexpansion States in Study Sample eFigure 2. Trends in Timely Prenatal Care by Race and Ethnicity and Nativity in Medicaid Nonexpansion States, 2011-2019 eTable 2. Sample Characteristics by Nativity, Nonexpansion States, Pre- and Post- Medicaid Expansion eTable 3. Sample Characteristics by Nativity Among Asian Women in Expansion Women in Expansion States Pre- and Post-Medicaid Expansion eTable 4. Sample Characteristics by Nativity Among Black Women in Expansion Women in Expansion States Pre- and Post-Medicaid Expansion eTable 5. Sample Characteristics by Nativity Among Hispanic Women in Expansion Women in Expansion States Pre- and Post-Medicaid Expansion eTable 6. Sample Characteristics by Nativity Among White Women in Expansion Women in Expansion States Pre- and Post-Medicaid Expansion eTable 7. Rate of Timely Prenatal Care Pre- and Post-Medicaid Expansion in Nonexpansion States, by Nativity and Race and Ethnicity, 2011-2019 eTable 8. Rate of Timely Prenatal Care Pre- and Post-Medicaid Expansion in Nonexpansion States, by Nativity and Race and Ethnicity, Among Women With High School Education or Less, 2011-2019 [file jamanetwopen-e2239264-s001.pdf]

Supplementary Online Content

Janevic T, Weber E, Howell FM, Steelman M, Krishnamoorthi M, Fox A. Analysis of state Medicaid expansion and access to timely prenatal care among women who were immigrant vs US born. *JAMA Netw Open*. 2022;5(10):e2239264. doi:10.1001/jamanetworkopen.2022.39264

eFigure 1. Flowchart of Study Sample Inclusion and Exclusion Criteria

eTable 1. Medicaid Nonpregnancy and Pregnancy Coverage for Immigrants Pre- and Post-Medicaid Expansion in Expansion and Nonexpansion States in Study Sample

eFigure 2. Trends in Timely Prenatal Care by Race and Ethnicity and Nativity in Medicaid Nonexpansion States, 2011-2019

eTable 2. Sample Characteristics by Nativity, Nonexpansion States, Pre- and Post-Medicaid Expansion

eTable 3. Sample Characteristics by Nativity Among Asian Women in Expansion Women in Expansion States Pre- and Post-Medicaid Expansion

eTable 4. Sample Characteristics by Nativity Among Black Women in Expansion Women in Expansion States Pre- and Post-Medicaid Expansion

eTable 5. Sample Characteristics by Nativity Among Hispanic Women in Expansion Women in Expansion States Pre- and Post-Medicaid Expansion

eTable 6. Sample Characteristics by Nativity Among White Women in Expansion Women in Expansion States Pre- and Post-Medicaid Expansion

eTable 7. Rate of Timely Prenatal Care Pre- and Post-Medicaid Expansion in Nonexpansion States, by Nativity and Race and Ethnicity, 2011-2019

eTable 8. Rate of Timely Prenatal Care Pre- and Post-Medicaid Expansion in Nonexpansion States, by Nativity and Race and Ethnicity, Among Women With High School Education or Less, 2011-2019

This supplementary material has been provided by the authors to give readers additional information about their work.

© 2022 Janevic T et al. *JAMA Network Open.*

eFigure 1. Flowchart of study sample inclusion and exclusion criteria

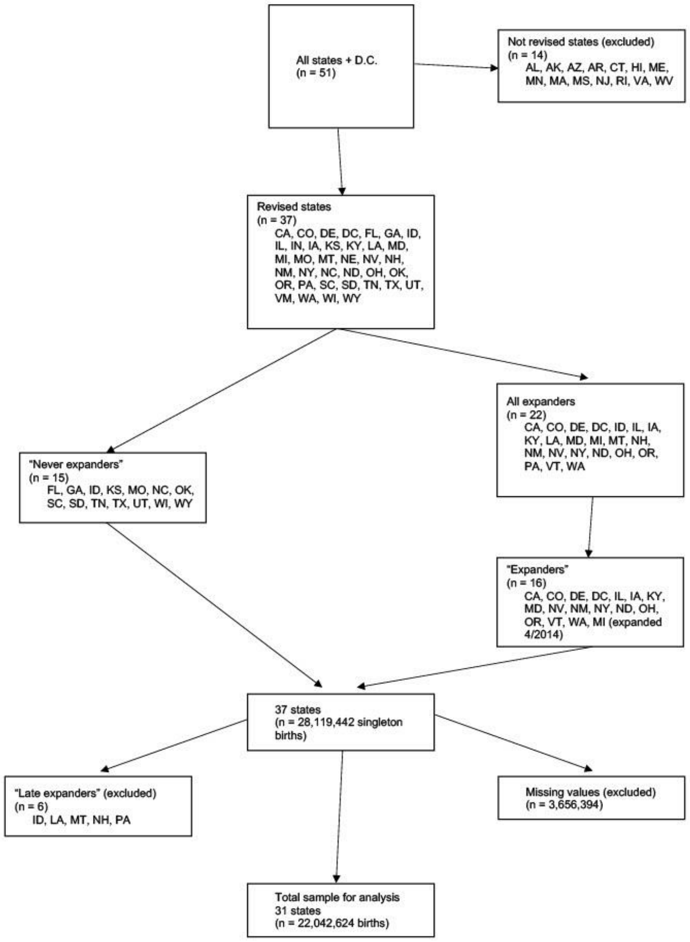

© 2022 Janevic T et al. *JAMA Network Open.*

eTable 1. Medicaid nonpregnancy and pregnancy coverage for immigrants pre- and post-Medicaid expansion in expansion[a] and nonexpansion[b] states in study sample

NON-PREGNANCY COVERAGE			PREGNANCY COVERAGE	
	Authorized Adult (non-pregnancy) coverage	Authorized Adult (non-pregnancy) coverage	Authorized Adult Pregnancy Coverage	Unauthorized Adult Pregnancy Coverage
	Medicaid for LPR[c] adults after 5 year bar[d]	Medicaid for LPR adults during 5 year bar[d]	Medicaid for LPR pregnant adults during 5 year bar[e]	State only funds to cover unauthorized pregnant adults[e]
**Yes**	CA, CO, DE, **FL**, **GA**, **ID**, IL, IA, **KS**, KY, MD, MI, **MO**, **NE**, NV, NM, NY, **NC**, OH, **OK**, OR, **SC**, **SD**, **TN**, **UT**, VT, WA, **WI**	CA, NY	CA, CO, DE, IL, MD, MI, **NC**, **NE**, NM, NY, OH, **OK**, OR, **TN**, **TX**, VT, WA, **WI**, **WY**	CA, IL, MI, **NE**, NY, **OK**, OR, **TN**, **TX**, WA, **WI**
**No**	ND, **TX**, **WY**	CO, DE, **FL**, **GA**, **ID**, IL, IA, **KS**, KY, MD, MI, **MO**, **NE**, NV, NM, **NC**, ND, OH, **OK**, OR, **SC**, **SD**, **TN**, **TX**, **UT**, VT, WA, **WI**, **WY**	**FL**, **GA**, **ID**, IA, **KS**, KY, **MO**, NV, ND, **SC**, **SD**, **UT**	CO, DE, **FL**, **GA**, **ID**, IA, **KS**, KY, MD, MI, **MO**, NV, NM, **NC**, ND, OH, **SC**, **SD**, **UT**, VT, **WY**

[a]16 expansion states included in study are noted in black: California, Colorado, Delaware, Illinois, Iowa, Kentucky, Maryland, Michigan, Nevada, New Mexico, New York, North Dakota, Ohio, Oregon, Vermont, and Washington

[b]15 non-expansion states in the study are noted in bolded red: Florida, Georgia, Idaho Kansas, Missouri, North Carolina, Nebraska, Oklahoma, South Carolina, South Dakota, Tennessee, Texas, Utah, Wisconsin and Wyoming
[c]LPR= Legal Permanent Resident

[d]Data on non-pregnancy coverage and unauthorized pregnancy coverage were accessed from the Urban Institute's State Immigration Policy Resource Data: https://www.urban.org/features/state-immigration-policy-resource

[e]Data on pregnancy coverage during the five-year bar were accessed from: KFF and the Georgetown University Center for Children and Families: https://www.kff.org/medicaid/report/annual-updates-on-eligibility-rules-enrollment-and-renewal-procedures-and-cost-sharing-practices-in-medicaid-and-chip/

There were no changes recorded in pregnancy or non-pregnancy coverage policies observed over the study period (2012-2020)

© 2022 Janevic T et al. *JAMA Network Open.*

eFigure 2. Trends in timely prenatal care by race and ethnicity and nativity in Medicaid nonexpansion states, 2011-2019

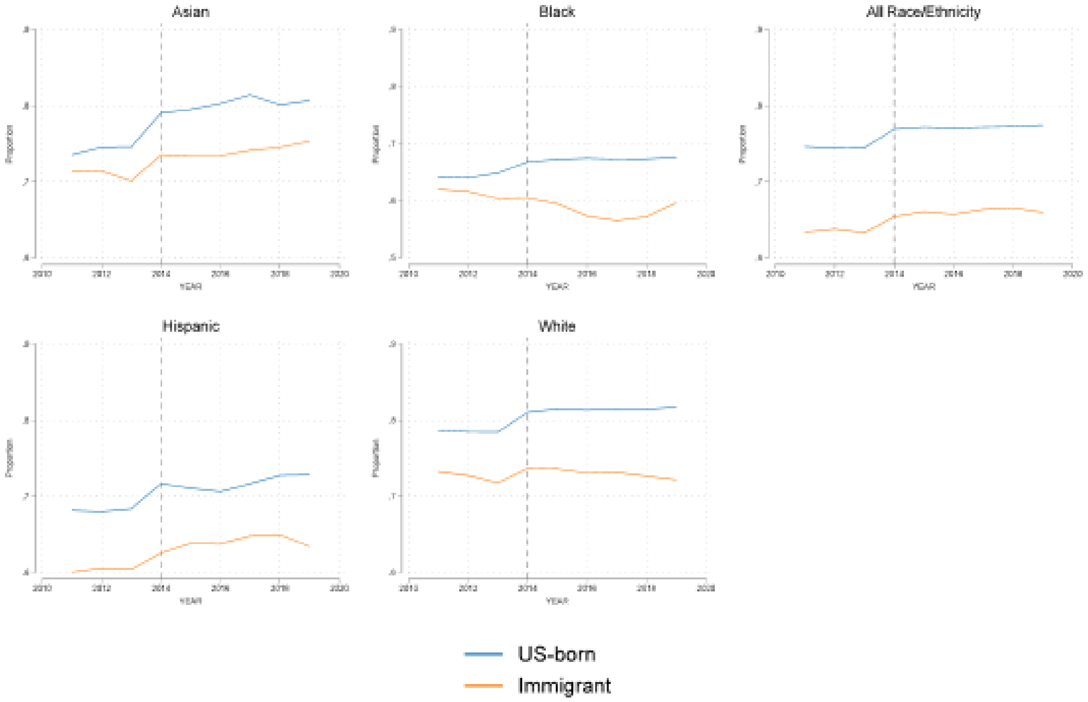

© 2022 Janevic T et al. *JAMA Network Open.*

eTable 2. Sample characteristics by nativity , nonexpansion states, pre- and post- Medicaid expansion

	Pre-expansion (2011-2014)		Post-expansion (2015-2019)	
**Characteristic**	**Immigrant n=945,866**	**US-Born n=3,492,213**	**Immigrant n=1,241,773**	**US-Born n=4,426,966**
Race-Ethnicity %				
Asian	145,740 (15.4)	28,079 (0.8)	198,515 (16.0)	36,600 (0.8)
Black	95,593 (10.1)	623,557 (17.9)	144,935 (11.7)	809,235 (18.3)
Hispanic	592,623 (62.7)	495,774 (14.2)	744,569 (60.0)	713,163 (16.1)
White	111,910 (11.8)	2,344,803 (67.1)	153,754 (12.4)	2,867,968 (64.8
Age Category %				
20-24	177,111 (18.7)	1,051,637 (30.1)	194,775 (15.7)	1,139,971 (25.8)
25-29	285,537 (30.2)	1,141,656 (32.7)	353,386 (28.5)	1,468,595 (33.2)
30-34	282,479 (29.9)	880,874 (25.2)	395,948 (31.9)	1,204,481 (27.2)
35-39	159,745 (16.9)	344,629 (9.9)	233,306 (18.8)	517,765 (11.7)
40-54	40,994 (4.3)	73,417 (2.1)	64,358 (5.2)	96,154 (2.2)
Multiparous %				
Yes	657,728 (69.5)	2,210,228 (63.3)	842,471 (67.8)	2,840,822 (64.2)
Education %				
< High School	316,054 (33.4)	321,890 (9.2)	329,781 (26.6)	344,026 (7.8)
High School	233,717 (24.7)	889,180 (25.5)	321,731 (25.9)	1,158,508 (26.2)
Some College	169,692 (17.9)	1,241,534 (35.6)	230,358 (18.6)	1,524,800 (34.4)
Bachelors / Graduate	226,403 (23.9)	1,039,609 (29.8)	359,903 (29.0)	1,399,632 (31.6)

© 2022 Janevic T et al. *JAMA Network Open.*

eTable 3. Sample characteristics by nativity among Asian women in expansion states, pre- and post- Medicaid expansion

	Pre-expansion (2011-2014)		Post-expansion (2015-2019)	
**Characteristic**	**Immigrant n=413,479**	**US-Born n=96,807**	**Immigrant n=520,560**	**US-Born n=122,147**
Age Category %				
20-24	29,903 (7%)	13,998 (14%)	29,208 (6%)	9,683 (8%)
25-29	110,926 (27%)	24,766 (26%)	131,144 (25%)	24,477 (20%)
30-34	156,739 (38%)	36,758 (38%)	205,142 (39%)	50,851 (42%)
35-39	92,422 (22%)	17,944 (19%)	123,546 (24%)	31,571 (26%)
40-54	23,489 (6%)	3,341 (3%)	31,520 (6%)	5,565 (5%)
Multiparous %				
Yes	223,507 (54%)	50,709 (52%)	280,032 (54%)	63,483 (52%)
Education %				
< High School	34,599 (8%)	2,715 (3%)	34,826 (7%)	1,760 (1%)
High School	56,689 (14%)	12,494 (13%)	65,034 (12%)	11,692 (10%)
Some College	78,254 (19%)	24,571 (25%)	88,954 (17%)	25,558 (21%)
Bachelors / Graduate	243,937 (59%)	57,027 (59%)	331,746 (64%)	83,137 (68%)

© 2022 Janevic T et al. *JAMA Network Open.*

eTable 4. Sample characteristics by nativity among Black women in expansion states, pre- and post-Medicaid expansion

	Pre-expansion (2011-2014)		Post-expansion (2015-2019)	
**Characteristic**	**Immigrant** **n=** 110,829	**US-Born** **n=** 470,128	**Immigrant** **n=** 150,570	**US-Born** **n=** 562,810
Age Category %				
20-24	13,176 (12%)	182,276 (39%)	14,806 (10%)	175,525 (31%)
25-29	30,139 (27%)	138,231 (29%)	38,410 (26%)	185,217 (33%)
30-34	36,246 (33%)	94,098 (20%)	51,238 (34%)	123,553 (22%)
35-39	23,639 (21%)	43,704 (9%)	34,532 (23%)	63,138 (11%)
40-54	7,629 (7%)	11,819 (3%)	11,584 (8%)	15,377 (3%)
Multiparous %				
Yes	71,901 (65%)	309,916 (66%)	100,510 (67%)	375,588 (67%)
Education %				
< High School	16,210 (15%)	67,198 (14%)	20,017 (13%)	63,902 (11%)
High School	27,582 (25%)	146,451 (31%)	37,410 (25%)	192,576 (34%)
Some College	34,721 (31%)	187,696 (40%)	43,703 (29%)	215,899 (38%)
Bachelors / Graduate	32,316 (29%)	68,783 (15%)	49,440 (33%)	90,433 (16%)

© 2022 Janevic T et al. *JAMA Network Open.*

eTable 5. Sample characteristics by nativity among Hispanic women in expansion states, pre- and post- Medicaid expansion

	Pre-expansion (2011-2014)		Post-expansion (2015-2019)	
**Characteristic**	**Immigrant** **n=** 752,176	**US-Born** **n=** 699,776	**Immigrant** **n=** 808,843	**US-Born** **n=** 965,214
Age Category %				
20-24	153,340 (20%)	267,934 (38%)	142,760 (18%)	308,142 (32%)
25-29	229,570 (31%)	211,087 (30%)	222,443 (28%)	322,796 (33%)
30-34	207,402 (28%)	147,776 (21%)	239,678 (30%)	214,585 (22%)
35-39	126,304 (17%)	60,829 (9%)	155,792 (19%)	99,999 (10%)
40-54	35,560 (5%)	12,150 (2%)	48,170 (6%)	19,692 (2%)
Multiparous %				
Yes	577,336 (77%)	447,211 (64%)	612,792 (76%)	616,151 (64%)
Education %				
< High School	367,829 (49%)	108,044 (15%)	329,595 (41%)	115,962 (12%)
High School	208,286 (28%)	223,476 (32%)	245,215 (30%)	315,659 (33%)
Some College	115,370 (15%)	263,634 (38%)	145,318 (18%)	370,480 (38%)
Bachelors / Graduate	60,691 (8%)	104,622 (15%)	88,715 (11%)	163,113 (17%)

© 2022 Janevic T et al. *JAMA Network Open.*

eTable 6. Sample characteristics by nativity among White women in expansion states, pre- and post- Medicaid expansion

	Pre-expansion (2011-2014)		Post-expansion (2015-2019)	
**Characteristic**	**Immigrant** **n=** 238,746	**US-Born** **n=** 2,608,873	**Immigrant** **n=** 307,543	**US-Born** **n=** 3,107,305
Age Category %				
20-24	28,928 (12%)	540,454 (21%)	31,027 (10%)	530,353 (17%)
25-29	66,515 (28%)	817,638 (31%)	79,383 (26%)	935,660 (30%)
30-34	79,681 (33%)	814,375 (31%)	107,959 (35%)	1,041,962 (34%)
35-39	49,690 (21%)	355,390 (14%)	69,632 (23%)	501,900 (16%)
40-54	13,932 (6%)	81,016 (3%)	19,542 (6%)	97,430 (3%)
Multiparous %				
Yes	139,633 (58%)	1,558,327 (60%)	184,307 (60%)	1,895,646 (61%)
Education %				
< High School	16,914 (7%)	159,532 (6%)	22,023 (7%)	171,387 (6%)
High School	40,397 (17%)	520,537 (20%)	49,355 (16%)	617,443 (20%)
Some College	58,444 (24%)	854,523 (33%)	68,120 (22%)	948,069 (31%)
Bachelors / Graduate	122,991 (52%)	1,074,281 (41%)	168,045 (55%)	1,370,406 (44%)

© 2022 Janevic T et al. *JAMA Network Open.*

eTable 7. Rate of timely prenatal care pre- and post-Medicaid expansion in nonexpansion states, by nativity and race and ethnicity, 2011-2019

	Pre				Post					
Race-ethnicity	Immigrant %	US-Born %	Adjusted[a] Diff[b] n per 100	95% CI	Immigrant %	US-Born %	Adjusted[a] Diff[b] n per 100	95% CI	Diff-in-Diff	95% CI
All	64	75.1	-6.44	(-8.69, -4.20)	66.3	77.3	-7.48	(-9.18, -5.77)	-1.03	(-2.16, 0.01)
Asian	71.6	75.4	-4.44	(-5.18, -3.70)	74.1	80.5	-5.92	(-6.57, -5.27)	-1.48	(-2.13, -0.84)
Black	61.1	64.7	-7.92	(-12.59, -3.25)	57.9	66.9	-12.83	(-19.24, -6.42)	-4.91	(-7.01, -2.82)
Hispanic	61	69.2	-6.12	(-8.73, -3.51)	64.6	71.9	-6.18	(-8.05, -4.32)	-0.06	(-1.04, 0.91)
White	72.8	79.1	-6.96	(-9.21, -4.72)	72.9	81.6	-9.14	(-10.30, -7.98)	-2.18	(-4.89, 0.54)

[a] Adjusted for race, age, parity, education, state unemployment rate, state poverty rate and Immigrant Climate Index
[b] Adjusted difference for Immigrant vs. US-Born

© 2022 Janevic T et al. *JAMA Network Open.*

eTable 8. Rate of timely prenatal care pre- and post-Medicaid expansion in non-expansion states, by nativity and race-ethnicity, among women with high school education or less, 2011-2019

	Pre				Post					
	Immigrant %	US-Born %	Adjusted[a] Diff[b] n per 100	95% CI	Immigrant %	US-Born %	Adjusted[a] Diff[b] n per 100	95% CI	Diff-in-Diff	95% CI
All	56.7	64.6	-8.04	(-10.30, -5.79)	59.8	67.1	-7.82	(-9.39, -6.24)	0.23	(-1.12, 1.57)
Asian	55.8	61.3	-8.45	(-11.73, -5.17)	59	67.3	-10.12	(-13.31, -6.94)	-1.67	(-4.39, 1.04)
Black	55.8	58.2	-5.83	(-8.67, -2.99)	52.9	60.8	-10.35	(-13.37, -7.33)	-4.52	(-5.47, -3.57)
Hispanic	56.5	62.3	-7.94	(-9.22, -6.66)	60.4	65.8	-7.57	(-8.56, -6.58)	0.37	(-0.39, 1.14)
White	62.5	68.3	-5.47	(-10.52, -0.42)	63.2	70.8	-6.92	(-9.36, -4.48)	-1.45	(-4.69, 1.79)

[a] Adjusted for race, age, parity, education, state unemployment rate, state poverty rate and Immigrant Climate Index
[b] Adjusted difference for Immigrant vs. US-Born

© 2022 Janevic T et al. *JAMA Network Open.*
